# A Multianalyzer Machine Learning Model for Marine Heterogeneous Data Schema Mapping

**DOI:** 10.1155/2014/248467

**Published:** 2014-08-28

**Authors:** Wang Yan, Le Jiajin, Zhang Yun

**Affiliations:** ^1^Glorious Sun School of Business and Management, Donghua University, Shanghai, China; ^2^College of Information Technology, Shanghai Ocean University, Shanghai, China; ^3^School of Computer Science and Technology, Donghua University, Shanghai, China

## Abstract

The main challenges that marine heterogeneous data integration faces are the problem of accurate schema mapping between heterogeneous data sources. In order to improve the schema mapping efficiency and get more accurate learning results, this paper proposes a heterogeneous data schema mapping method basing on multianalyzer machine learning model. The multianalyzer analysis the learning results comprehensively, and a fuzzy comprehensive evaluation system is introduced for output results' evaluation and multi factor quantitative judging. Finally, the data mapping comparison experiment on the East China Sea observing data confirms the effectiveness of the model and shows multianalyzer's obvious improvement of mapping error rate.

## 1. Introduction 

### 1.1. Heterogeneity of Marine Data and Integration Mappings

Marine data is typical heterogeneous data with multiscale, multitemporal, and multisemantic features. Marine data fields include marine resource, marine geography, marine biology, marine chemistry, and many other fields.

Due to the difference between acquisition equipment, information processing platforms, and data storage formats, even in the same field heterogeneous data still exist. In schema mapping, marine heterogeneous data's features are described as follows.

1. Large scale: large-scale marine data come from marine large monitor and sensor systems which transmit huge amount of marine data to data center.
2. Distribution uncertainty: distribution uncertainty includes both physical distribution and logical distribution uncertainty. The physical distribution uncertainty is that the data source is not physically concentrated but distributed in multiregions, connected by network between pluralities of data sources. And logical uncertainty is that different properties may even exist in a same data cube's different data fields.
3. Semantic heterogeneity: it means rules and data types in each marine data source are more or little different. And there is also data semantic heterogeneity in marine databases. Different granularity division, different entities relations, and entities semantic description difference result in heterogeneous semantic data.
4. Semistandardized: although schema and data's semantic heterogeneity exist in marine database, the descriptions of marine information have some certain specifications and standards. These loose constraints are called semistandardized.

Marine heterogeneity is an organic integration of marine data with different formats, source, and characteristics logically or physically. The integration goal is to convert, adjust, decompose, and merge marine data's different formal features (such as units, format, and scale) and internal features (such as property) and form compatible seamless marine data sets finally [1]. In the process of marine data integration, how to improve the efficiency of heterogeneous data schema mapping should be considered.

Schema mapping relation is the combination of elements mapping between heterogeneous data models in the same field or heterogeneous island. Schema mapping problem is a research hotspot in heterogeneous data integration. Schema mapping is a process that users select data sets by operate the data interface, determine the data flow direction, and select target database and mapping table. How to implement automatic or semiautomatic schema mapping is the problem of machine mapping.

**Figure 1:**
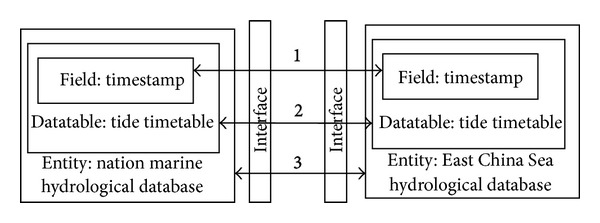
Digital ocean ETL mapping hierarchy.

In Figure 1, arrows 1–3, respectively, represent three types of scheme mapping: model mapping, table mapping, and field mapping. Marine heterogeneous data mapping relationship is divided into these three levels. Model mapping described by one or more attributes sets reflects the conceptual model difference of heterogeneous database mapping. Table mapping is an entity mapping process basing on the corresponding table relation. The across table mapping relation can be simplified as one-one mapping. Field mapping is an attribute mapping in a relational database and it is the underlying mapping relationship. Mapping process firstly extracts concept model from the entire heterogeneous data sources and then abstracts, classifies, and refines the correspondence relationship between source and destination and forms the mapping model finally. We take marine field schema heterogeneous difference between marine observation stations as an example to explain the characteristics of heterogeneous data exchange in Figure 8.

### 1.2. Automatic Schema Mapping Based on Machine Learning

Automatic mapping for heterogeneous data can be achieved by learning machine. A large number of researches indicate multistrategy machine learning usually is more accurate than single one. While it increases the complexity of the system, a lower error rate can be obtained.

Currently, the researches of multi-strategy machine learning focus on learning model combination. In [2, 3] BayesIDF learning method and grammar learning method are combined effectively, and a multi-strategy approach is proposed for information extraction. The multistrategy learning model in [4] refers to the framework of the result combination of multiple learning methods, which is known as meta-learning [5]. These analyses theoretically prove that learning result combination is more accurate than the best results of the individual learner. Rule-based model and maximum entropy model are combined in [6] to provide a hybrid approach of determining an appropriate time contact between a pair of entities. A new multi-domain online learning framework based on parameters is proposed in [7]. Ensemble matrix is proposed in [8] which can help users understand the relative merits of various classifiers and analyzers and allows users to explore and establish a communication model by direct visualization. Some other integration methods are presented recently, such as boosting [9], stacking [10], and bagging [11]. All these methods repeat single analyzer training, apply the results to different parts of one problem, and combine these results to obtain performance improvement.

Because of the huge differences between marine heterogeneous data, it is more difficult to determine and give an accurate judgment for the individual output of the learner. In order to evaluate output results of the automatic schema mapping more accurately, multianalyzer concept is put forward in this paper, and then the fuzzy comprehensive evaluation is applied to multianalyzer model to quantify various factors of learner's output during heterogeneous sampling and get more accurate learning results combination. Finally, the multianalyzer model is verified in multisource marine observation data mapping experiments.

## 2. Automatic Schema Mapping Based on Machine Learning

**Figure 2:**
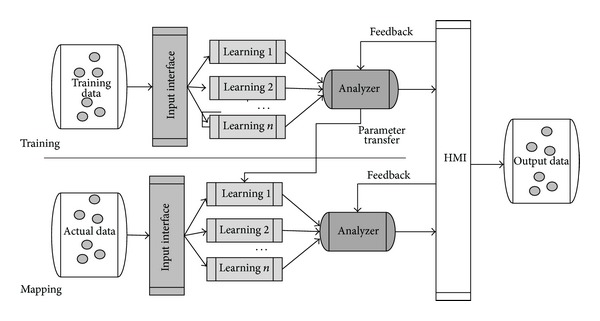
Flow diagram of automatic schema mapping of machine learning.

Automatic mapping model of machine learning generally contains input interface, learner, analyzer, and human interface as shown in Figure 2. The input interface checks, filters, and selects sample data. Learner is responsible for statistical analysis and feature extraction. After analyzing the learning results, the analyzer can adjust the parameters of the learning machine and reconfigure the strategies to improve the schema mapping efficiency and real-time matching. Users operate UI and input feedback information and parameters to form positive feedback by human interface.

During training phase, training data are set by default; learning machine extracts the parameters of data sets; and then internal schema mapping prediction model is generated. The analyzer analyzes output of learning machine and feedbacks parameters. During schema mapping phase, according to the prediction model, the learner analyzes data source and monitors schema mapping results dynamically. In self-learning process, the selection of learning sample is an important part. Sample sets should be of the maximum global matching attributes. There is sample property set *β*, and  *β* = {*b*_1_, *b*_2_,…, *b*_*n*_}. And global data samples *γ* have global properties data set {*b*_*f*1_, *b*_*f*2_,…, *b*_*fn*_}. When min⁡ ({*b*_1_, *b*_2_,…, *b*_*n*_}, {*b*_*f*1_, *b*_*f*2_,…*b*_*fn*_}), sample set *β* can be called the best sample set.

For marine data system, assuming there are two kinds of data, source and target, we can establish schema mapping from source and target. For example, there are sites, latitudes, and longitudes information from sensor nodes as marine source data. After the target schema field and specified schema relationship are defined, a training process table in detail can be obtained.

If the samples are extracted according to the probability distribution model, probability distribution of the large sample could be calculated and data selection function of input interface could be optimized. Though many samples are required, the training process cannot cover all the instance sets. If there is a probability distribution of statistical samples, property study can be obtained in advance.

**Figure 3:**
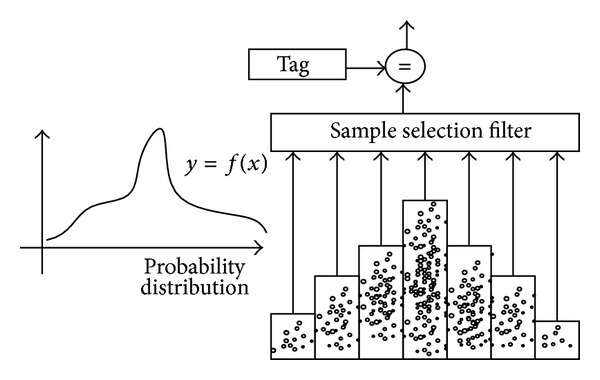
Sample filter based on the probability distribution.

When the sample data sets are selected, attribute tags for sample data can be previously identified, and these tags are recorded in the real environments, which can mark the distribution density of the attribute, as shown in Figure 3.

Samples can be searched according to tags in high probability distribution interval and sorted according to probability distribution. We can get various property data sets, including highly matched sets, moderate matched sets, and lowly matched ones. Samples in various property data sets are selected randomly and the training results then are modified by manual intervention interface during training.

According to the probability distribution, learning results are transferred into analyzers, function modules in the analyzer feedback the judgment information, inquiring learner's pre-judgments for training. The analyzer's field judgments can be mathematically expressed as follows:

(1) p=m(f(xs,p,t),xd)={1,p<0.4  (Default  probability  1  threshold)0,p≥0.8  (Default  probability  0  threshold).

**Figure 4:**
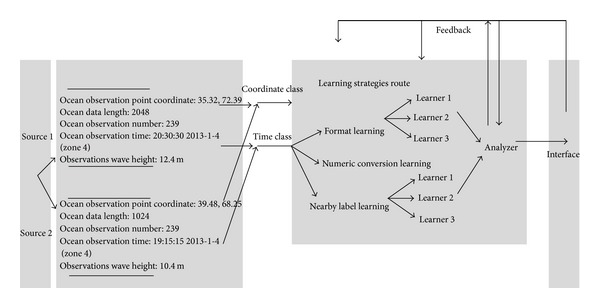
Machine schema mapping process of marine observation data.

For any instance of group *x*_*s*_, we get schema mapping values by schema mapping function *f* and compare these values to the known target instances; 0 or 1 will be obtained by calculating matching probability and comparing them to the probability thresholds. Then, learner gives the scores of the schema mapping. If there is multilearner, multiple learners' output needs to be compared and ranked. Finally, the analyzer will output record tables, which cover sample properties and the parameters proposals, and dynamically adjust the parameters according to current input and responses. After training, analyzer can begin mapping process for new data sources. A marine observation data mapping process is shown in Figure 4.

The matching distribution probability data are extracted from observation source and used for detecting and judging the information unit. Then, the correlation matching probability of learning machine is given for 0/1 judgment according to predefined threshold value. During the whole process, users give the real-time feedback corrections by operating UI.

But method of single analyzer is not fitful to judge and analyze data from multiple dimensions of learners. The output data of learner are of multidimensional attribute, and their attributes may be orthogonal. So, analyzer for multidimension becomes important. Multistudy strategies are used to capture the pattern of association between schemas and improve the matching accuracy for the whole system. But single analyzer is not fitful for the combination of multistudy strategies, and multianalyzer strategies can provide convenience for multistudy strategies parsing. Calculation and analysis of single analyzer often take a long time, and multianalysis reduces the analysis time by parallel computing. In this paper, multianalyzer is put forward to increase the dimensions of analysis and improve the matching schema mapping.

## 3. Heterogeneous Data Schema Mapping Optimization Basing on Multianalyzer

### 3.1. Multianalyzer Concept

The analyzer can select the best learning path from the output of the learners; so, analyzing theme domain subdivision, major subject identification, and their universality, completeness, and independency should be taken into consideration. So, the selected subject field should cover other subject fields. Secondly, evaluation granularity affects the difficulty and parallelism of judgment and calculation. Finally, the selection of the appropriate evaluation method must base on samples feature.

**Figure 5:**
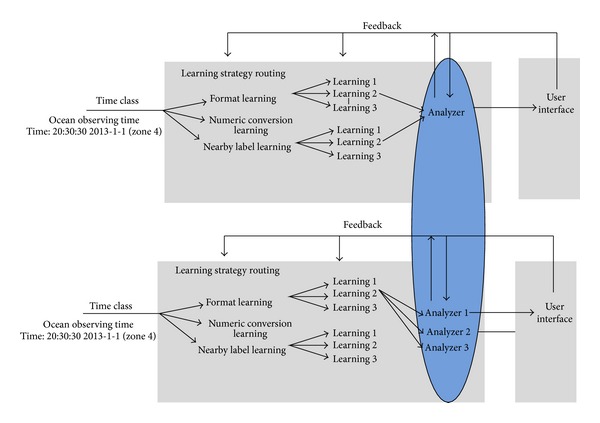
Multianalyzer automatic schema mapping structure.

In automatic schema mapping model, multianalyzer will improve the schema mapping accuracy. In Figure 5, multianalyzer will build a score matrix for the matching correlation of the learner's results.

Multi-analyzer can expand match and synthesis, introduce multi-stage matching and integration, and select the best analysis results from the output.

Field analysis can be expressed as a multidimensional function to extend the determination mode shown as below. *m*_*i*_ is the determination function, with mapping function *f*. Consider

(2) $$\begin{array}{c} P_{n}=\left(\begin{array}{c} m_{1}\left(f\left(x_{s},p,t\right),x_{d}\right)\\ m_{2}\left(f\left(x_{s},p,t\right),x_{d}\right)\\ m_{3}\left(f\left(x_{s},p,t\right),x_{d}\right)\\ \vdots \\ m_{n}\left(f\left(x_{s},p,t\right),x_{d}\right) \end{array}\right). \end{array}$$

But, for multidimensional learning results, how to convert them to single analysis output and how to select an appropriate function to achieve dimension reduction processing and simplify the analysis are difficult and important. There are huge differences in massive samples and heterogeneous data, so the final output and determination of multi-analyzer be more difficult and complicated. So the fuzzy comprehensive evaluation is introduced for multi-analyzer's result to simplify the result by the fuzzy dimension reduction.

### 3.2. Fuzzy Comprehensive Evaluation Method

The basic idea of fuzzy comprehensive evaluation is to consider the various factors associated with objects and make a reasonable evaluation with the fuzzy linear transformation theory and the maximum membership degree principle.

There are *m* factors related to target of evaluation, the set of these factors is called as the factor set and denoted as below:

(3) $$\begin{array}{c} U=\left\{u_{1},u_{2},\ldots ,u_{m}\right\}. \end{array}$$

There are *n* comments which are called evaluation set and recorded as below:

(4) $$\begin{array}{c} V=\left\{v_{1},v_{2},\ldots ,v_{m}\right\}. \end{array}$$

Firstly, each factor *u*_*i*_ in factors set *U* is single evaluation factor which determines the membership degree value *r*_*ij*_ of the reviews *v*_*j*_ and forms a single-actor evaluation set of *u*_*i*_ as

(5) $$\begin{array}{c} r_{i}=\left(r_{i1},r_{i2},\ldots ,r_{in}\right),\;r_{i}\in \mu \left(V\right). \end{array}$$

So, we can get fuzzy sets of the reviews set *V*. Then, a fuzzy-value schema mapping function is shown below as

(6) $$\begin{array}{c} f:U⟶F\left(V\right),\;\;u_{i}⟶f\left(u_{i}\right), \end{array}$$

where *f*(*u*_*i*_) = *r*_*ij*_ = (*r*_*i*1_, *r*_*i*2_,…, *r*_*in*_) is the comments fuzzy vector on the factors set *u*_*i*_ and *r*_*ij*_ is the relationship factor between *u*_*j*_ and *v*_*j*_.

Then, all the single factor evaluation factors are integrated together to build a general evaluation of matrix and a fuzzy comprehensive evaluation matrix *R* from *U* to *V* as follows:

(7) $$\begin{array}{c} R=\left(\begin{array}{cccc} r_{11} & r_{12} & \cdots & r_{1n}\\ r_{21} & r_{22} & \cdots & r_{2n}\\ \cdots & \cdots & \cdots & \cdots \\ r_{m1} & r_{m2} & \cdots & r_{mn} \end{array}\right). \end{array}$$

In other words, fuzzy value function gets the relationship from *U* to *V*, where *R*_*f*_ ∈ *F* (*U* × *V*);  *R*_*f*_ is the comprehensive evaluation matrix.

As the effects of all the factors for evaluation are different, strong or weak, a fuzzy weight factor set is necessary for matrix *U*, which is defined below as

(8) $$\begin{array}{c} A=\left(a_{1},a_{2},\ldots ,a_{m}\right), \end{array}$$

where *a*_*i*_ is the measure of the impacts on the evaluation for the factors *u*_*i*_  (*i* = 1,2, ..., *m*), and *A* is called importance fuzzy subset on the matrix *U*, and *a*_*i*_ is called importance coefficient for factor *u*_*i*_.

Then, fuzzy comprehensive evaluation model is given to calculate the fuzzy comprehensive evaluation set. When the factors of importance fuzzy sets *A* and comprehensive evaluation matrix (fuzzy relations) *R* are known, fuzzy subsets *A* on evaluation matrix *V* are got by linear transform from *R* as

(9) bj=(a1∗∧r1j)∗∨(a2∗∧r2j)∗∨⋯∗∨(am∗∧rmj),(j=1,2,…,n),

where $\overset{\wedge }{*}$ indicates generalized fuzzy “and” operation, and $\overset{\wedge }{*}$ indicates generalized fuzzy “or” operation. And *B* is called as fuzzy comprehensive evaluation set for *V*, and the formula is known as the comprehensive evaluation model, and denoted as $M\left(\overset{\wedge }{*},\overset{\vee }{*}\right)$.

Finally, according to the maximum principle of membership degree, the maximum largest membership *b*_*j*_ in the set (*b*_*l*_, *b*_2_,…, *b*_*n*_) is the result of comprehensive evaluation, where review element *v*_*j*_ is corresponded to *b*_*j*_ in fuzzy comprehensive evaluation set.

Compared to single factor *u*_*i*_, the membership factor of evaluation *u*_*i*_ for review factor *v*_*j*_ is *r*_*ij*_ (*j* = 1, 2,…, *n*), while the results of operations $\left(a_{i}\overset{\wedge }{*}r_{ij}\right)$ (denoted by *r*_ij_*) can reflect all the influence factors and obtain more accurate evaluations, that is, the fuzzy comprehensive evaluation method's merit.

### 3.3. Multianalyzer with Fuzzy Comprehensive Evaluation

In order to evaluate the learner's output, during the process of output quantization, fuzzy comprehensive evaluation method can be applied to learning machine for massive heterogeneous marine data schema mapping and takes all dynamic, ambiguous, and real-time factors into account. In the schema mapping system with multianalyzer, fuzzy comprehensive evaluation method will improve automatic map's accuracy and sensitivity.

(1) The evaluation factors set of learning model is *U* = {*U*_1_, *U*_2_,…, *U*_*n*_}; the weight set is *A* = {*A*_1_, *A*_2_,…, *A*_*n*_}; and *A*_*i*_ is the weight of evaluation factor *U*_*i*_, 0 ≤ *A*_*i*_ ≤ 1,  ∑_*i*=1_^*n*^ *A*_*i*_ = 1.

And *U*_*i*_ = {*U*_*i*1_, *U*_*i*2_,…, *U*_*in*_}; *n* is the number of specific performance factor basing on design factors. The weight set *A*_*i*_ = {*a*_*i*1_, *a*_*i*2_,…, *a*_*in*_}, where *a*_*ij*_ is the weighing of *u*_*ij*_, where 0 ≤ *a*_*ij*_ ≤ 1,  ∑_*j*=1_^*n*^ *a*_*ij*_ = 1.

The performance factors set of the automatic schema mapping learning machine model is shown in Table 1.

Weight set is determined by the way of matching degree evaluation according to the expert points-scoring system's evaluation for all kind of performance indexes.

The single factor performance evaluation of learning model evaluates the membership index of each factor, and a fuzzy schema mapping function is given as *£* : *U* → *V*.

For each *U*_*i*_, *R*_*i*_ can be shown as the fuzzy matrix as follows:

(10) $$\begin{array}{c} R_{i}=\left\{r_{ik}\right\}=\left(\begin{array}{cccc} r_{11} & r_{12} & \ldots & r_{1n}\\ r_{21} & r_{22} & \ldots & r_{2n}\\ \ldots & \ldots & \ldots & \ldots \\ r_{m1} & r_{m2} & \ldots & r_{mn} \end{array}\right), \end{array}$$

where *r*_*jk*_ represents the membership grade of factors *u*_*ij*_ to the *k* level evaluation *V*_*k*_. The value of *r*_*jk*_ can be determined by expert points-scoring system. The number of *v*_*i*_ level reviews for *u*_*ij*_ is *s*_*i*_; so, we get

(11) $$\begin{array}{c} r_{jk}=\frac{S_{k}}{\sum_{j=1}^{m}S_{k}}. \end{array}$$

The membership vector *B*_*i*_ of the evaluation set *V*, *B*_*i*_ = *A*_*i*_ × *R*_*i*_ = (*b*_*i*1_, *b*_*i*2_,…, *b*_*im*_), is the summary result of performance single factor fuzzy evaluation result on the factor *U*_*i*_.

After the single factor evaluation, fuzzy comprehensive evaluation can extend the evaluation to all levels learning indexes and constitute fuzzy matrix *B* by a single factor *B*_*i*_. One has

(12) $$\begin{array}{c} B=\left(\begin{array}{c} B_{1}\\ \cdots \\ B_{n} \end{array}\right)=\left(\begin{array}{c} b_{11}\cdots b_{1m}\\ b_{n1}\cdots b_{nm} \end{array}\right). \end{array}$$

And after the fuzzy performance matrix operations to *R*, the membership vector *B* is given for factors set *U* to comment *V* level as

(13) $$\begin{array}{c} B=A\times R=\left(b_{1},b_{2},\ldots ,b_{m}\right). \end{array}$$

If ∑_*j*=1_^*m*^ *b*_*j*_ ≠ 1, the normalization expression is

(14) $$\begin{array}{c} {\overset{\wedge }{b}}_{j}=\frac{b_{j}}{\sum_{j=1}^{m}b_{j}}. \end{array}$$

According to the principle of maximum membership, *V*_*k*_ is the performance level element in model design stage which is closely corresponded with the maximum membership degree *b*_*k*_ in *B*. The decision-maker determines the performance index of the model design According to the critical index threshold. It should be noted that the weight of the model needs to be adjusted for the different learning machine models and learning strategies. So, for multilearning strategies, weight parameters sets may be loaded dynamically.

## 4. Experiments

We compare the mapping accuracy of single analyzer's output with multianalyzer using fuzzy comprehensive evaluation for 20 sets of data with different information format which come from different marine observation stations in the East China Sea. And, for a single analyzer, we use both the statistical analyzer and format analyzer. The analyzer's description is shown in Table 2.

The factors and weights of fuzzy comprehensive evaluation can be adjusted according to the actual requirements during the marine heterogeneous data schema mapping process. For example, in the process of multisource marine tidal data, the evaluation factors and the weights range are shown in Table 3.

**Figure 6:**
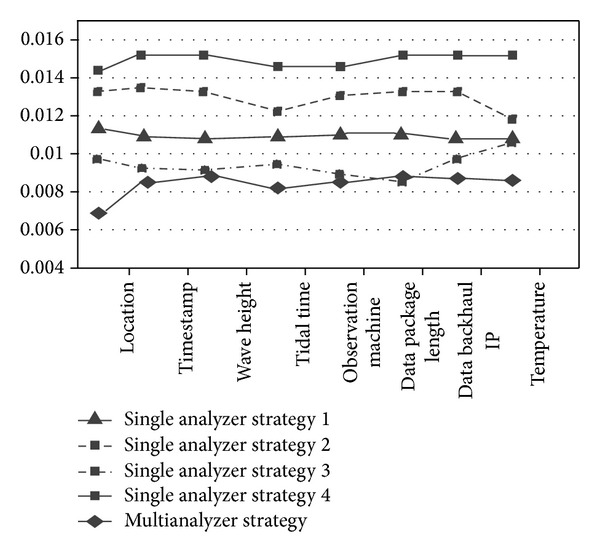
Comparative analysis strategy of single learner.

**Figure 7:**
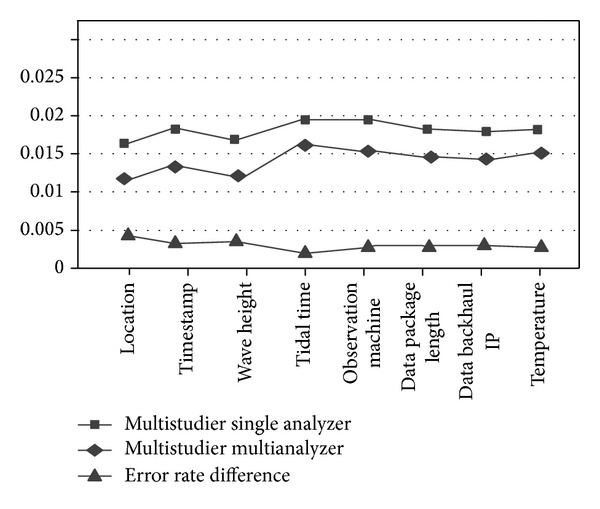
Comparative analysis of multilearner strategies.

**Figure 8:**
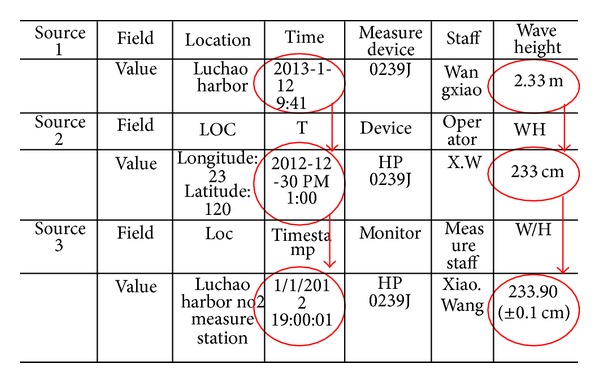
Marine heterogeneous data field format difference.

We compare single analyzer's output results with multianalyzers for the East China Sea marine observatory stations' heterogeneous data. The *x*-axis is field information: location, time, wave height, tide time, observation instrument information, data packet length, data transmission IP address, and temperature. And we define the mapping error rate to measure mapping quality for data mapping which is the ratio of total error number and total mapping data number. The experiments results for different analysis strategies for the marine test data sets are obtained in Figures 6 and 7.

In Figure 6, single strategy multianalyzer machine learning results' mapping error rate is 17.6% less than that of the best single analyzer (numerical analyzer), and it is 28.5% less than that of probability statistical analyzer. In addition, multianalyzer's process time is 9% less than single analyzer's process time for parallel process. In Figure 7, average mapping error rate of multistrategy multianalyzer machine learning is 25.7% less than that of multistrategy single analyzer, and the process time is reduced by 7%.

## 5. Conclusion

Learning machine model is the research hotspot of automatic data schema mapping. Because of marine data's heterogeneity, large capacity, and multiple dimensions, the outputs of single analyzer become too difficult to judge accurately. In order to process multidimension learning data, provide convenience for multistudy strategies parsing, and reduce the process time, this paper presents the concept of multianalyzer learning machine and uses fuzzy comprehensive evaluation method to evaluate multioutput. So, various factors can be combined and parallel-processed to evaluate learning machine's results more effectively and accurately. And marine data's multidimensions and heterogeneity can be taken into consideration to improve the schema mapping.

More test configurations of study strategy and analysis strategy may produce interesting results, though we expect multi-analyzer to maintain a performance advantage if computer resource is sufficient. Detailed analysis of hardware structure and process schedule of multi-analyzer would also be useful to improve the result output.

## Figures and Tables

**Table 1:** Evaluation factor hierarchical table.

Objective	Level 1	Level 2
Evaluation of learner's output	Correlation model evaluation *U*_1_	Correlation model evaluation *U*_11_
		Correlation model evaluation *U*_12_
		⋮
	Performance analysis evaluation *U*_2_	Performance analysis evaluation *U*_21_
		Performance analysis evaluation *U*_22_
		⋮
	Adaptability analysis evaluation *U*_3_	Adaptability analysis evaluation *U*_31_
		Adaptability analysis evaluation *U*_32_
	⋮	⋮

**Table 2:** List of analyzers' type.

	Analyzer	Descriptions
1	Format and numerical analyzer	Judge whether the mapping results conforms to rules of format and value
2	Time analyzer	Statistics and analysis of learning, analysis, and mapping time
3	Statistical analyzer	Scoreboard for study results based on prior probability
4	Correlation coefficient and stability analyzer	Calculating data's Pearson correlation coefficients in same field and analyzing the stability basing on cycle time

**Table 3:** Tidal data evaluation factors hierarchy partition.

Objective	Level 1	Level 2	Weights
Evaluation of multisource marine tidal data's learning machine output	Correlation model evaluation	Conversion coefficient score of the outputs of tide data between *t*_1_ and *t*_*n*_	15%~20%
		Matching score of same dimensions tidal data (surface data/underwater data) conversion output	10%~15%
		Output distribution probability matching of key data (dynamic location coordinates, time information, wave height information, and tide time information)	5%~10%
		Tide height limit spatial matching degree	0%~5%
	Conversion performance analysis evaluation	Response time (max, min, and avg) under typical long term observation	5%~10%
		Response time (max, min, and avg) under typical short term observation	5%~10%
		Response time (max, min, and avg) under long term observation tidal day	0%~5%
		Response time (max, min, and avg) under short time observation tidal day	5%~10%
	Adaptability analysis evaluation	Data fluctuation under long term observation	5%~10%
		Output fluctuation of same dimensions (surface data/underwater data) tidal data	5%~10%

## References

[1] Dongmei H, Chi Z, Jipeng D (2012). Integration of massive multi-source heterogeneous space-time data in digital sea. *Marine Environmental Science*.

[2] Michalski RS, Carbonell JG, Mitchell TM (1985). *Machine Learning: An ArtificialIntelligence Approach*.

[3] Domingos P (1996). Unifying instance-based and rule-based induction. *Machine Learning*.

[4] Freitag D (2000). Machine learning for information extraction in informal domains. *Machine Learning*.

[5] Chan PK, Stolfo SJ Experiments on multistrategy learning by meta-learning.

[6] Chang YC, Dai HJ, Wu JCY (2013). TEMPTing System: a hybrid method of rule and machine learning for temporal relation extraction in patient discharge summaries. *Journal of Biomedical Informatics*.

[7] Dredze M, Kulesza A, Crammer K (2010). Multi-domain learning by confidence-weighted parameter combination. *Machine Learning*.

[8] Talbot J, Lee B, Kapoor A EnsembleMatrix : interactive visualization to support machine learning with multiple classifiers.

[9] Freund Y, Schapire RE Experiments with a new boosting algorithm.

[10] Wolpert DH (1992). Stacked generalization. *Neural Networks*.

[11] Breiman L (1996). Bagging predictors. *Machine Learning*.

